# Randomised, double-blind, placebo-controlled study investigating the effects of inorganic nitrate on vascular function, platelet reactivity and restenosis in stable angina: protocol of the NITRATE-OCT study

**DOI:** 10.1136/bmjopen-2016-012728

**Published:** 2016-12-20

**Authors:** Krishnaraj S Rathod, Daniel A Jones, T J A Van-Eijl, Hilda Tsang, Helen Warren, Stephen M Hamshere, Vikas Kapil, Ajay K Jain, Andrew Deaner, Neil Poulter, Mark J Caulfield, Anthony Mathur, Amrita Ahluwalia

**Affiliations:** 1 Barts NIHR Cardiovascular Biomedical Research Unit, Centre of Clinical Pharmacology, William Harvey Research Institute, Queen Mary University, London, UK; 2 Department of Cardiology, Barts Heart Centre,2 St. Bartholomew's Hospital, Barts Health NHS Trust, London, UK; 3 Imperial Clinical Trials Unit, Imperial College, London, UK; 4 King George Hospital, Barking and Havering NHS Trust, London, UK

## Abstract

**Introduction:**

The mainstay treatment for reducing the symptoms of angina and long-term risk of heart attacks in patients with heart disease is stent implantation in the diseased coronary artery. While this procedure has revolutionised treatment, the incidence of secondary events remains a concern. These repeat events are thought to be due, in part, to continued enhanced platelet reactivity, endothelial dysfunction and ultimately restenosis of the stented artery. In this study, we will investigate whether a once a day inorganic nitrate administration might favourably modulate platelet reactivity and endothelial function leading to a decrease in restenosis.

**Methods and design:**

NITRATE-OCT is a double-blind, randomised, single-centre, placebo-controlled phase II trial that will enrol 246 patients with stable angina due to have elective percutaneous coronary intervention procedure with stent implantation. Patients will be randomised to receive 6 months of a once a day dose of either nitrate-rich beetroot juice or nitrate-deplete beetroot juice (placebo) starting up to 1 week before their procedure. The primary outcome is reduction of in-stent late loss assessed by quantitative coronary angiography and optical coherence tomography at 6 months. The study is powered to detect a 0.22±0.55 mm reduction in late loss in the treatment group compared with the placebo group. Secondary end points include change from baseline assessment of endothelial function measured using flow-mediated dilation at 6 months, target vessel revascularisation (TVR), restenosis rate (diameter>50%) and in-segment late loss at 6 months, markers of inflammation and platelet reactivity and major adverse cardiac events (ie, myocardial infarction, death, cerebrovascular accident, TVR) at 12 and 24 months.

**Ethics and dissemination:**

The study was approved by the Local Ethics Committee (15/LO/0555). Trial results will be published according to the CONSORT statement and will be presented at conferences and reported in peer-reviewed journals.

**Trial registration numbers:**

NCT02529189 and ISRCTN17373946, Pre-results.

**Strengths and limitations of this study**

- This is the first randomised-controlled trial assessing the use of dietary nitrate to reduce the rates of restenosis in patients undergoing percutaneous coronary intervention for stable angina.
- This study will determine the potential of dietary nitrate as adjunctive therapy in patients with stable angina.
- This study will determine whether a sustained elevation of nitrite, using a safe method of administration, might cause a decrease in the extent of restenosis following stent implantation and in this way reduce the need for repeat intervention.
- This is a single-centre study; therefore, the applicability of the results to other units would be affected.

## Introduction

Coronary heart disease is the single most common cause of death in the UK causing 1 in 7 and 1 in 10 deaths in men and women, respectively (http://www.bhf.org.uk). Presently, timely percutaneous coronary intervention (PCI) with stent implantation remains the most effective treatment strategy for limiting events and infarct size following an acute myocardial infarction, preserving left ventricular ejection fraction and improving clinical outcomes.^1–4^ However, despite these advances,^3–5^ substantial mortality^6^ and morbidity rates persist with respect to longer term outcome. In simple lesions, restenosis rates have been estimated to be <5% at 1 year but at 5 years, repeat intervention rates are ∼10%.^7^ However, in more complex lesions, restenosis has been documented at 10% within 2 years.^8^ In addition, recent assessments of patients undergoing PCI (∼50% with stable angina) demonstrate that in-stent thrombosis, despite antiplatelet therapy, remains a concern, with recent calls urging identification of more effective and safer antiplatelet therapy.^9^ ^10^ A major determinant of prognosis after treatment is the reocclusion of the affected arteries. A number of specific phenomena have been linked with reocclusion including persistent endothelial dysfunction, increased platelet reactivity and restenosis. Therefore, strategies that might limit or indeed correct these phenomena have clear therapeutic potential.

In the healthy cardiovascular system, tonically generated nitric oxide (NO), produced via the conventional L-arginine/NO synthesis pathway, plays an essential role in maintaining homoeostasis and in sustaining healthy cardiac function, perfusion and cardioprotection.^11^ ^12^ In patients with coronary artery disease, a generalised ‘endothelial dysfunction’ which is characterised by deficient endothelium-derived bioavailable NO exists; the extent of which is correlated with severity of coronary artery disease.^13^ The cardioprotective effects of NO relate to a number of actions including its potent vasodilator effect in the ischaemic myocardium,^14^ allowing for essential perfusion of injured tissue, its anti-inflammatory effects repressing leucocyte recruitment,^15^ its antiplatelet effects^14^ ^16^ ^17^ and its antiproliferative influence over vascular smooth muscle.^14^ ^18^ Thus, the replacement of this ‘lost’ NO represents an approach that might offer therapeutic utility.

A potential solution for elevating endogenous NO levels lies in the chemical reduction of inorganic nitrite (NO_2_^−^) to NO. Indeed, nitrite-derived NO protects against myocardial ischaemia/reperfusion injury in preclinical models,^19^ ^20^ attenuates vascular smooth muscle cell proliferation in a model of balloon injury in rats,^21^ protects against endothelial dysfunction^22^ ^23^ and attenuates platelet reactivity in healthy volunteers and patients with hypercholesterolaemia.^24^ ^25^ Importantly, NO_2_^−^ does not suffer tachyphylaxis and its function is not dependent on metabolising enzymes that are dysfunctional in cardiovascular disease as with organic nitrates; factors that are major limiting issues underlying the difficulties with organic nitrates experienced within the clinical setting.^26^

Recently, a simple and safe method has been identified for elevating circulating nitrite levels via inorganic nitrate supplementation through consumption of vegetables (eg, beetroot). In healthy volunteers and patients with cardiovascular disease dietary nitrate, in the form of beetroot juice, causes dose-dependent rises in circulating nitrite levels associated with improvement in endothelial dysfunction, and suppressed platelet reactivity assessed ex vivo.^23–25^ ^27^ Furthermore, there have now been several studies in patients demonstrating the benefits of inorganic nitrate, administered either through the use of nitrate-rich beetroot juice or through provision of a nitrate salt dose, with respect to improvements in maximal oxygen consumption and diastolic function in heart failure^28^ ^29^ as well as reduction in platelet reactivity and improvements in endothelial function in patients with hypercholesterolaemia^25^ that importantly could have direct relevance for patients with angina undergoing elective intervention.

Thus, together all of the above issues support the rationale for further exploration of potential approaches based on elevation of circulating nitrite levels that might reduce the burden of revascularisation on the National Health Service (NHS), and underline the rationale for the clinical trial described here.

## Methodology

### Trial objectives

*Aims of research*: The trial is designed to test whether dietary nitrate ingestion in addition to conventional therapy has beneficial effects in patients with stable angina. In particular, the aim is to investigate whether dietary nitrate leads to antiplatelet effects, improvement of endothelial function and improvements of intimal hyperplasia thereby possibly resulting in reductions of restenosis rates post-PCI and stent implantation.

### Participant selection

This is a single-centre trial and patients will be recruited at Barts Health NHS Trust within the Barts and the London Heart Attack Centre, based at the Barts Heart Centre, St Bartholomew's Hospital. In addition, we will identify patients from King George Hospital which is part of Barking, Havering and Redbridge NHS Trust.

### Original hypothesis

Dietary nitrate ingestion, in addition to conventional therapy, improves vascular function in patients with stable angina undergoing PCI.

Specifically, the aims of the study are:

1. To determine whether dietary nitrate might improve intimal hyperplasia and thereby restenosis rates post-PCI and stent implantation.
2. To determine whether dietary nitrate ingestion exerts antiplatelet effects or improvement of endothelial function and the mechanisms involved in this effect.

### Primary end point

The primary end point will be reduction of in-stent late loss assessed by angiography (quantitative coronary angiography, QCA) at 6±1 months. Assessment of restenosis will be made by measurement of in-stent late loss assessed by angiography and optical coherence tomography (OCT) at 6±1 months.

### Secondary end points

1. Improvement in endothelial function assessed by flow-mediated dilation (FMD) of the brachial artery at 6 months compared with preprocedure assessment.
2. Reduction in target vessel revascularisation (TVR), restenosis rate (diameter >50%) and in-segment late loss at 6±1 months.
3. Reduction in major adverse cardiac events (ie, myocardial infarction, death,) at 6, 12 and 24 months in addition to cardiovascular accident and TVR.
4. Reduction in plaque size as assessed using OCT at 6±1 months.
5. Reduction in inflammatory markers and changes in plasma xanthine oxidoreductase activity, high-sensitivity C reactive protein, interleukin-6 at 6 and 12 months.
6. Reduction in platelet aggregation ex vivo at 6 and 12 months compared with preprocedure.

### Inclusion criteria

1. Patients with stable angina diagnosed by a cardiologist on optimal medical therapy undergoing angioplasty to treat residual symptoms.
2. Aged 18–85.
3. Patients able and willing to give their written informed consent.
4. Patients undergoing successful PCI procedure.

### Exclusion criteria

1. Unstable ischaemic heart disease, with an episode of chest pain in <24 hours before inclusion into the study.
2. Patients who have had previous coronary artery bypass surgery, if they are undergoing angioplasty within a non-native vessel.
3. Patients undergoing angioplasty with a bioabsorbable stent since the stents will likely start dissolving prior to 6 months and therefore this would confound measures of restenosis.
4. Current diagnosis of, or treatment for, malignancy other than non-melanoma skin cancer.
5. Current life-threatening condition other than vascular disease that may prevent a participant completing the study.
6. Use of an investigational device or investigational drug within 30 days or five half-lives (whichever is the longer) preceding the first dose of study medication.
7. Patients considered unsuitable to participate by the research team (eg, due to medical reasons, laboratory abnormalities or participant's unwillingness to comply with all study-related procedures).
8. Severe acute infection, or significant trauma (burns, fractures).
9. Pregnancy tested by urine human chorionic gonadotropin measurement.
10. History of alcohol or drug abuse within the past 6 months.
11. A history of heart failure New York Heart Association (NYHA) class 3–4 or severe left ventricular dysfunction left ventricular ejection fraction <30% regardless of symptom status.
12. Systemic autoimmune disease such as rheumatoid arthritis, connective tissue disease or other conditions known to be associated with chronic inflammation such as inflammatory bowel disease.
13. Patients who have donated >500 mL blood within 56 days prior to study medication administration.
14. Anaemia with haemoglobin <10 g/dL, or any other known blood disorder or significant illness that may affect platelet function, and coagulation.
15. A history of chronic viral hepatitis (including presence of hepatitis B surface antigen or hepatitis C antibody or other chronic hepatic disorder) or HIV.
16. Abnormal liver function due to acute or chronic liver conditions 3×upper limit of normal at screening.
17. Renal impairment with creatinine clearance (estimated glomerular filtration rate) of 35 mL/min at screening.
18. If patients are on mouthwash, they must be willing to stop using this at least 1 week before the start of the study and throughout the duration that they are involved in the study.

### Study design and intervention

**Figure 1:**
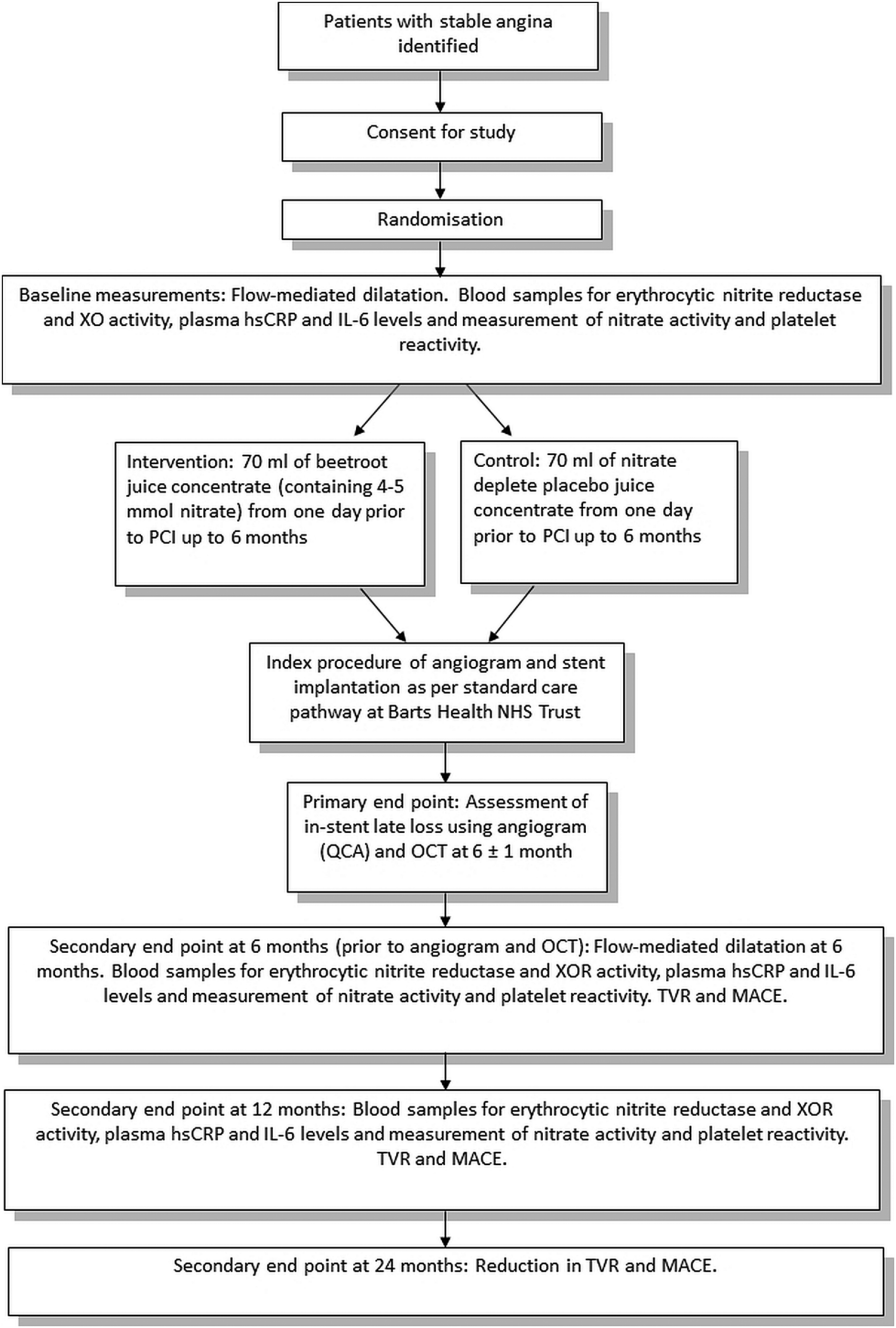
hsCRP, high-sensitivity C reactive protein; IL-6, interlukin 6; MACE, major adverse cardiac events; NHS, National Health Service; OCT, optical coherence tomography; PCI, percutaneous coronary intervention; QCA, quantitative coronary angiography; TVR, target vessel revascularisation; XOR, xanthine oxidoreductase.

This is a prospective double-blind, placebo-controlled, clinical study. A total of 246 patients (male and female, age 18–85) with stable angina as per requirements indicated above will be recruited. Figure 1 shows a summary of the study scheme. Since patients with diabetes are at high risk of developing restenosis, these patients will also be included in our study with stratification in both groups. Patients will be stratified according to the type of stent (ie, bare metal stent (BMS) or drug-eluting stent (DES)) as both these groups have differing characteristics resulting in differing rates of restenosis. Follow-up will take place in the Clinical Trials Unit, William Harvey Heart Centre. The patients will inevitably have other comorbidities that include raised blood pressure and hypercholesterolaemia, and these will be recorded. Patients will be block randomised (using an online randomisation database) to receive 70 mL of a beetroot juice concentrate containing ∼5 mmol nitrate or nitrate-depleted placebo juice concentrate (James White Drinks, UK) control. The volunteers will start taking their daily dose at home the day before the scheduled angioplasty and continue daily for 6 months. Patients will be advised to take their dose of juice at the same time each day, preferably in the morning with their breakfast. Patients will also be provided with dietary advice in relation to the calorific content of the juice: a daily dose of 70 mL of the juice concentrate (∼70 g) contains about 100 kcal.

On the day of index procedure, patients will be treated as per Barts Health Trust protocols. Here, the patient will arrive to the day case ward in the morning of the procedure and the senior interventional trainee registrar and consultant cardiologist will perform the coronary angiogram. At the time of the procedure, the operator will determine the route of access (either via the radial artery or femoral artery) and the consultant cardiologist will determine the size (width and length) and type of stent (DES or BMS) that will be inserted. Following the procedure, the patient will be transferred back to the day case ward and discharged by either the nurse in charge or the cardiology registrar depending on the complexity of the procedure. Patients will be followed up according to the clinical plan suggested by the consultant cardiologist at the time of the procedure.

### Randomisation and blinding process

Patients will be block randomised on a 1:1 basis to receive either dietary nitrate or placebo, using a binary random number sequence (http://www.random.org). Treatment assignment for volunteers in the dietary nitrate and placebo groups will remain blinded until data lock and statistical analysis at the end of the study. If unblinding is required, the chief investigator for the study will be informed. A list of the unblinded treatments will be kept in a secure location at the the William Harvey Heart Centre. The unblinding procedure will be available at all times (24 hours day/7 days a week).

### Methods to be used

#### Blood, saliva and urine analysis

In this study, blood samples will be taken for the assessment of platelet reactivity, and NO level determination from the venous side of the circulation from an arm vein using a yellow butterfly needle (19 gauge) while the participant lies on a comfortable couch. In this study protocol on visit 2, they will have one such blood test before starting the treatment or placebo juice. They will have a further blood test at visit 3 (6 months—prior to angiogram and OCT) and visit 5 (12 months). Blood samples will be centrifuged immediately for platelet reactivity experiments and plasma and red blood cell storage only for the purposes of making biochemical measurements (eg, nitrate/nitrite/cyclic guanosine monophosphate) and will be discarded once used.

Saliva and urine will be collected in a falcon tube at the time of visits and stored for purposes of measuring nitrate/nitrite and then discarded. Some saliva will be centrifuged and a pellet generated. This pellet contains oral bacteria that have dislodged from the oral cavity. This pellet will be frozen for identification and analysis of the oral microbiota by second-generation genome sequencing at a later date.

The nitrate/nitrite concentrations in saliva, blood and urine will be determined using the technique of chemiluminescence as previously described.^30^ In brief, total nitrate and nitrite concentration (termed ‘NO_x_’) will be determined by adding samples to 0.1 mol/L vanadium (III) chloride in 1 M hydrochloric acid refluxing at 95°C under nitrogen. Nitrite concentration will be determined by the addition of samples to 0.09 mol/L potassium iodide in a glacial acetic acid under nitrogen at room temperature. Nitrate concentration will be calculated by the subtraction of (nitrite) from (NO_x_). All measurements of all samples will be conducted by an individual blinded to the intervention.

#### Pulse wave analysis and pulse wave velocity

This technique is a non-invasive method to measure arterial stiffness. A Vicorder device (Skidmore Medical Limited, Bristol, UK) will be used to simultaneously record the pulse wave from the carotid and femoral site using an oscillometric method. A small, inflatable neck pad will be placed directly over a single carotid artery and secured around the neck by a Velcro tab. A cuff will be placed around the patient's ipsilateral upper thigh. Both carotid and femoral cuffs will be inflated automatically to 65 mm Hg and the corresponding oscillometric signal from each cuff will be digitally analysed to extract the pulse time delay. The distance between the sternal notch and the thigh cuff will be measured and used as a standard estimate for the aortic length. From these measurements, aortic pulse wave velocity (PWV) can be derived as PWV=aortic distance/pulse time delay.^31^

These procedures are not uncomfortable and take ∼10 min to complete and will be conducted at visit 2 (baseline), visit 3 (6 months—prior to angiogram and OCT) and visit 5 (12 months).

#### Index angiogram

Angiography will be performed at baseline prior to stent placement and after stent placement prior to completion of the index PCI procedure. Either the radial or femoral vascular access may be used for the procedure.

The baseline angiograms of the involved vessel will be performed in at least two near orthogonal views. Visual angiographic assessment will be used to determine if the lesion meets angiographic entry criteria.

Further information collated from the index coronary angiography procedure will be collected, comprising of date of the angiogram, vessel dominance, number and location of diseased vessels, location, presence and severity of lesions for all 14 segments of the coronary tree and any complications. The PCI procedure will be completed according to normal practice by the operator.

#### Index PCI

Procedural information for the index PCI will include date of the PCI, which coronary vessels and segments are treated, diameter, length and type of stent implanted, the procedural success for each segment, the adjunctive pharmacological therapy and any complications and information on lesions not treated will also be recorded.

#### Repeat coronary angiography with OCT assessment of vessel treated PCI

Repeat angiography with OCT will occur 6±1 months after index PCI. Either radial or femoral access sites can be used and angiograms of the involved vessel performed in at least two near orthogonal views. Quantitative angiographic assessment of the involved vessel will be undertaken to determine late loss. In addition, the same angiographic images will be recorded as those obtained post-stent implantation at the time of the index procedure.

#### Quantitative coronary angiography

Quantitative analysis will be performed using a Coronary Angiography Analysis System (Medis, the Netherlands). There will be automated detection of the boundaries of a selected coronary vessel segment using digitised and optically magnified sections of the cine frame. If a wrong centreline is chosen because of large daughter branches, then correction is possible manually. The vessel diameter will be calculated in absolute values (mm). This will be done using the boundaries of a section of the contrast catheter and comparing the computed mean catheter diameter in pixels with the known catheter size in mm. To detect the contours of the vessel, the user will indicate the vessel by choosing two centre positions proximal and distal to the area of interest. QCA will be analysed in a blinded fashion by two experienced and independent cardiologists and adjudicated by a senior cardiologist if there are any discrepancies in the analysis.

All angiographic measurements of the target lesion will be obtained in the ‘in-stent’ zone, within 5 mm proximal and distal to each stent edge, and over the entire segment (‘in-segment’ zone). The following QCA parameters will be calculated: reference vessel diameter, minimal lumen diameter, per cent diameter stenosis (difference between the reference vessel diameter and minimal lumen diameter/reference vessel diameter×100) and late lumen loss (difference between the postprocedure and follow-up minimal lumen diameter). Binary restenosis will be defined as stenosis of 50% or greater in the target lesion or segment at the 6-month angiographic follow-up.

#### Optical coherence tomography

OCT imaging of the previously stented segment with motorised pull back at 1 mm/s will be undertaken in all patients at 6±1 months after index PCI. OCT images will be acquired at 15–30 frames per second (500 angular pixels×250 radial pixels), from a 0.15 mm micro-optic core, 0.36 mm image wire, displayed with an inverse gray-scale lookup table, and digitally archived. OCT images will be analysed by an independent expert in high-resolution OCT analysis. An automatic analysis with novel dedicated software will be used for quantitative assessment. The existence of malapposition will be assessed. The OCT variables recorded will be endothelial coverage: expressed as per cent of struts without coverage, neointimal thickness at 6±1 months and stent malapposition defined as struts with detachment from the vessel ≥110 μm. Finally, OCT imaging will not be used for clinical decision-making regarding need for a repeat revascularisation procedure. All repeat revascularisation decisions will be based on a recurrence of clinic symptoms and/or non-invasive imaging evidence of myocardial ischaemia and made by the treating physician.

#### Flow-mediated dilation

Non-invasive ultrasound assessment of endothelial function of the brachial artery in participants will be determined by measurement of FMD. FMD is a non-invasive method of assessing endothelial function in vivo. It uses vascular ultrasound to measure the increase in the diameter of the brachial artery in response to increased flow^32^ and will be conducted according to published guidelines.^33^ Brachial artery diameter in the right arm will be measured with a high-resolution external vascular ultrasound Siemens\Acuson Sequoia C256 Colour Doppler with a 7.0-MHz linear-array transducer supported by a stereotactic clamp. The vessel will be scanned in longitudinal section and the centre will be identified when the clearest views of the anterior and posterior artery walls have been obtained. Images will be magnified with a resolution box function and images of the brachial artery acquired continuously using semiautomated edge detection software (FMD Studio) and analysed in real time. Here, an automatic mathematical contour tracking operator locates and tracks the edges, supplying information about quality and time course of measurements in real time.^34^ Blood flow velocity in the brachial artery will be recorded continuously throughout the study with pulsed-wave Doppler. Brachial artery diameter and blood flow velocity will be measured continuously for 1 min at baseline, during 5 min of reduced blood flow (induced by inflation to 300 mm Hg of a pneumatic cuff placed at a site distal to the segment of artery being analysed), and for a further 5 min during reactive hyperaemia after cuff release. FMD is defined as the maximum percentage increase in vessel diameter during reactive hyperaemia. This procedure will be performed at visit 2 (baseline) and visit 3 (6 months—prior to angiogram and OCT).

#### Follow-up

After 2 years, the participant will be contacted by telephone for assessment of major adverse cardiac events (MACE). The whole duration of the study will be 2 years. Participants have no obligation to complete the whole study and if they decide to withdraw at any point then they are free to do so.

### End of study definition

The study will end after 2 years after the telephone follow-up of the last patients. At this time, all the samples will be analysed.

### Statistical analysis

*Sample size*: In order to achieve an 80% probability that the study will detect a treatment difference at a two-sided 5% significance level, if the true difference in late loss between the treatments is 0.22 mm, sample size calculations determined that a total of 246 patients will be required to enter a two-treatment parallel design study. This absolute difference is calculated from a mean late loss of 1.27 mm with an SD of the response variable of 0.550. These values being the mean and average of the SDs of 22 trials measuring late loss in drug-eluting and BMSs described in the review by Mauri *et al*.^35^ Recruitment also takes into account an additional 10% to account for drop-out or withdrawal/non-compliance. This value is based on previous experience in our unit.

The sample size of 246 enables sufficient power for estimation of the hard end point of MACE at 6 months. Very recently, in patients with stable angina who have undergone elective angioplasty, a remote ischaemic preconditioning intervention resulted in a significant reduction in MACE at 6 months with 4/110 (3.6%) in the treatment group versus 13/104 (12.5%) in the control group.^36^ Using these data as a basis for power calculations, a total number of 230 patients are needed for 80% power using one-tailed analysis. Thus, to account for potential loss to follow-up, we will recruit 246 patients in total.

Power calculations for FMD suggest that the above numbers provide sufficient power for detecting differences in FMD (n=60). To calculate power, we used data provided in dietary studies including one demonstrating improvements in FMD following 6 weeks of artichoke juice,^37^ a further chronic study with walnuts^38^ and one meta-analysis^39^ assessing the effects of chronic polyphenol dietary interventions published in 2012. Calculating an averaged improvement of FMD of 1.1% with no change in the control and an averaged SD of 1.45 (determined from the average of the above studies) a total of 30 volunteers would be needed in each group requiring a total of 60 volunteers. Using our own data with dietary nitrate in patients with hypercholesterolaemia increases of FMD of 1% with an SD of 1.5%,^25^ a total of 74 patients are needed for sufficient power. If we assume a potential 10% drop-out rate, a total of 80 patients are required.

All statistical analyses will be conducted by the trial statisticians supported by the imperial clinical trials unit. Data will be analysed on an intention-to-treat basis. We will also conduct further per-protocol analyses and a subgroup analysis on patients who are on organic nitrates as part of their routine therapy, patients on statin therapy and a comparison of DESs versus BMSs by incorporating these variables in a Cox regression analysis model.

### Ethical considerations

The study protocol and any subsequent amendments, along with any accompanying material provided to the patient, in addition to any advertising material, was submitted by the investigator to an independent Research Ethics Committee (REC). Written approval from the Committee was obtained and subsequently submitted to the sponsor to obtain final sponsorship approval and NHS permissions.

#### Safety considerations

The intervention is 70 mL of a beetroot juice concentrate or nitrate-free placebo juice (James White Drinks, UK). The nitrate is extracted using the same extraction technique used to remove inorganic nitrate from the general drinking water supplies. There are no known harmful side effects from these interventions and this nitrate-free juice is not considered to be a drug or medicine and is classified as a foodstuff. In addition, several recent publications using the placebo juice are now available.^40–42^ In the unlikely event of an adverse event (AE) occurring directly as a result of the intervention, the trial would be stopped.

#### Safety reporting

An AE if not defined as serious, will be documented in the participants' medical notes (where appropriate) and the case report form (CRF) and followed up by the research team. Any serious adverse events (SAEs) that occur will be reported to the sponsor and main REC where in the opinion of the chief investigator the event was either ‘related’ (ie, it resulted from administration of any of the research procedures) and ‘unexpected’ (ie, the type of event is not listed in the protocol as an expected occurrence).

SAEs that are considered to be ‘related’ and ‘unexpected’ will be recorded in the participants' notes, the CRF, the sponsor SAE form and reported to the research office of the Trust within 24 hours of research staff being notified, and to the main REC within 15 days. The co-investigators in this study will be authorised to sign the SAE forms in the absence of the principal investigator. Since this study will be blinded, the treatment code for the patient will be broken in the reporting of an ‘unexpected and related’ SAE. This will be performed by an individual who is independent of the study and will allow the rest of the research team to remain blinded. The unblinding of single cases by the principal investigator in the course of this study will only be performed if necessary for the safety of the trial participant.

### Monitoring

#### Trial Steering Committee

The Trial Steering Committee (TSC) is composed of three independent experts in the fields of: pharmacology, interventional cardiology and clinical trials along with the investigators and the data monitor and medical statistician and one lay member. This committee has met before patient recruitment and will meet annually to assess safety, feasibility or any other arising problems (eg, with recruitment) and their recommendations will be followed.

#### Data Safety and Monitoring Board

An independent Data Safety and Monitoring Board (DSMB) has been formed to monitor patient safety as the study progresses. The DSMB has been selected by and communicates directly to the study's TSC. The committee includes a consultant cardiologist, an interventional cardiologist and a statistician. The DSMB met prior to initiation of the clinical study, after the recruitment of 10 patients and will meet at three monthly intervals. The DSMB will have access to unblinded patient data. If a serious concern with the safety of the patients in the trial arises, the DSMB may recommend early termination of the study.

### Dissemination

The study will be performed in agreement with the Declaration of Helsinki and is approved by the Local Ethics Committee (NRES Committee City Road and Hampstead: 15/LO/0555). Data collection will be completed by mid-2018. Primary and secondary analysis will start immediately after data monitoring is completed; publications will be prepared for submission in late 2018. The results of the trial will be published according to the CONSORT statement. Dissemination of results will focus on publications in peer-reviewed journals, presentations at national/international cardiology meetings and NHS groups. In accordance with recommendations, we have registered NITRATE-OCT with public registries, the UK Clinical Research Network (Study ID 20060), http://clinicaltrials.gov (NCT02529189) and Current Controlled Trials (International Standard Randomised Controlled Trials No: 17373946).

## Summary

There have been no clinical studies investigating the role of orally ingested nitrate in reducing restenosis in patients undergoing PCI for stable angina. NITRATE-OCT study is the first clinical study assessing the safety and efficacy of oral nitrate in a dietary form (beetroot juice) in this group of patients. This study will determine the potential of dietary nitrate as adjunctive therapy in patients with stable angina.
